# Mesoscale Modeling of Polymer Concrete Dynamic Properties

**DOI:** 10.3390/polym15214311

**Published:** 2023-11-02

**Authors:** Paweł Dunaj

**Affiliations:** Department of Mechanical Engineering and Mechatronics, West Pomeranian University of Technology in Szczecin, al. Piastów 19, 70-310 Szczecin, Poland; pawel.dunaj@zut.edu.pl

**Keywords:** mesoscale modeling, finite element method, substructural identification, polymer concrete, mineral casting, heterogenous material, damping

## Abstract

There is a constant need to predict the dynamic properties of composite materials already at the design stage. A particularly attractive tool for achieving this goal is mesoscale finite element modeling. This paper presents the mesoscale modeling of the dynamic properties of polymer concrete. The method is based on finite element modeling and substructural identification. Substructural identification is a model updating technique based on frequency response functions. It enables the identification of model dynamic properties considering damping. The presented method is used to model the dynamic properties of a polymer concrete beam. In the first step, the mesoscale finite element model is built and then it is decoupled into substructures: a polymer matrix, aggregates, and an interfacial transition zone (ITZ). Next, the dynamic properties of the polymer matrix substructure are updated, and the model is reassembled. Then, second-stage updating takes place, which consists of determining the parameters of the aggregates and the ITZ. The use of substructural identification made it possible to determine the parameters of substructures that do not exist in an independent, isolated form like the ITZ. Moreover, it allows for determining the amount of damping that ITZ brings to the structure.

## 1. Introduction

Epoxy polymer concrete is a heterogeneous, multi-phase composite consisting of various aggregates and epoxy resin as a binder [1]. Its properties largely depend on the type and proportion of the aggregates used [2,3,4]. The effect of the type and proportion of individual aggregates on the properties of polymer concrete can be evaluated at the material design stage [5,6,7,8]. For this purpose, mesoscale finite element models [9] and discrete element models are used [10]. 

Ma et al. [11] presented a three-phase finite element model of epoxy polymer concrete. The model was developed to describe the mechanical behavior of a material containing a large volume of irregularly shaped aggregates. The proposed model included granite aggregates, epoxy matrix, and the interfacial transition zone. The interfacial transition zone defined between the aggregates and matrix was considered to be the epoxy matrix, with its deformation highly restricted due to strong bonding to the aggregates. The developed mesoscale model was verified experimentally through a four-point bending test. The results show high agreement of the model with its real counterpart.

Song et al. [12] presented a topological deformation method for creating three-dimensional granular material models. The aggregates were modeled as star solids, which, according to the authors, better reflect their real counterparts than particle models of spheres, ellipsoids, and convex polyhedrons. Using a parametric description of the star solid, it was possible to precisely control the thickness of the interfacial transition zone. The proposed modeling method was validated against three-dimensional parameterized aggregate models of concrete. This led to the conclusion that the proposed method can effectively model a composite material with enhanced particles.

Zhou et al. [13] presented a comprehensive procedure for building a fully three-dimensional mesoscale finite element model for concrete-like materials. The model was aimed at obtaining a realistic representation of the real shapes and sizes of aggregate particles. In the proposed approach, three-dimensional polyhedral-shaped aggregates were represented by a convex hull. The mesoscale model generated from the proposed procedure was verified against standard experimental observations under quasi-static compression and tension. The simulation results were shown to be very close to the corresponding experimental observations.

Jin et al. [14] presented a three-dimensional mesoscale numerical model that can consider concrete heterogeneities and interactions between carbon fiber-reinforced polymer and concrete. The mesoscopic heterogeneities of concrete were regarded as a three-phase heterogeneous composite consisting of aggregate particles, mortar matrix, and interfacial transition zones. The coarse aggregate particles were assumed to be spherical, and they were modeled using two equivalent values (the first equivalent diameter of aggregates was 12 mm, and the second was 30 mm). Small coarse aggregate particles (about 5 mm) and fine aggregate particles (less than 5 mm) were equivalent to the mortar matrix. After comparing the established model with the experimental results, the authors stated that reasonable results in the failure mode and shear bearing capacity of the beam had been obtained.

Zhang et al. [15] developed a random aggregate model to simulate the mechanical behavior of steel fiber-reinforced concrete. The proposed model considered the mesoscale heterogeneity of steel fiber-reinforced concrete with explicitly modeled fibers. Steel fiber-reinforced concrete was considered a composite material consisting of four phases, i.e., coarse aggregate, mortar, and interfacial transition zones between the aggregate, mortar, and steel fibers. Good agreement between the simulation results and test observations indicated the validity of the proposed mesoscale numerical model.

Ouyang and Chen [16] proposed concrete meso-modeling based on the local background grid method. Random polyhedral aggregates were generated according to their gradation and individually placed directly in the background grid. According to the shape of the aggregate and its spatial location, the newly placed aggregate was encapsulated by a bounding box, in which the identification of concrete meso-components and detection of the intrusion of new and old aggregates were carried out. By transforming aggregate intrusion detection during the process of concrete meso-geometric modeling into overlap checking of aggregate elements in the local background grid, many disjoint conditions between new and old aggregates were avoided. Based on the developed model, compression and penetration simulations were carried out and experimentally verified. The results show that the model can qualitatively explain the influence of the meso-structure on the macro-mechanical behavior of concrete.

Maleki et al. [17] developed a finite element model of concrete at the mesoscale to investigate the effect of *ITZ* thickness on the tensile behavior of concrete. The finite element model was built in two stages. In the first, a concrete meso-structure was created, while aggregate particles with specific geometric shapes were generated based on a prescribed particle size distribution and randomly placed in a sample. In the second, a finite-element mesh including the meso-structure was generated. Validation and verification of the proposed meso-structural models of concrete were performed against published experimental and computational results, respectively. 

Naderi et al. [18] presented a method of modeling the fracture process in concrete under static and dynamic tensile loading considering its mesoscale structure. A three-dimensional meso-structure of concrete consisting of coarse aggregates, mortar, and the interfacial transition zone between them was developed using an in-house code based on the Voronoi tessellation [19] and splining method. This enabled the generation of realistic-look aggregates with controllable structural features such as content, location, size, and shape. A cohesive zone model was then employed to simulate the tensile fracture behavior of concrete in terms of stress- and energy dissipation–displacement responses and crack mechanisms against the shape (spherical and irregular) and volume fraction of the aggregate and the strain rate. The developed model was verified by the experimental results of the tensile stress–displacement response of the concrete specimen under static loading, giving consistent results. A similar modeling approach was used in [20].

Niknezhad et al. [21] presented a realistic morphological 3D mesoscale model composed of aggregate grains, cement mortar, and interfacial transition zones. Linear-elastic-brittle behavior for the aggregates and no influence of the *ITZ* on the overall compressive behavior of the concrete specimen were assumed in the model. The elasto-plastic mechanical behavior of the mortar was modeled using a concrete damage plasticity model [22]. Based on the developed model, a series of virtual tests was conducted to characterize the mechanical behavior under quasi-static loading, as well as the transport properties of cementitious composite. Tests included the effect of the shape, volume fraction, and segregation of inclusions (coarse aggregates) on these properties.

Mazzucco et al. [23] presented the mesoscale modeling of concrete with recycled aggregates. To obtain a realistic geometric representation of recycled aggregates, a few single samples of aggregates were laser scanned. Based on the scanned geometry, a solid model was established. Next, the method of radial distances was implemented to reproduce a realistic volume fraction of a concrete sample made with recycled aggregates. The proposed modeling method was shown to correctly represent and describe local confinement effects, as well as the real inelastic behavior of concrete samples.

Wu et al. [24] developed random 3D aggregate models that feature rough surfaces and sharp angular shapes to accurately mimic coarse-grained aggregates in steel-fiber-reinforced concrete. In addition, straight, hooked-end, and spiral steel fiber models were created and randomly inserted into these aggregate models. The developed mesoscale models were verified using experimental compression test data available in the literature, and then used for simulation and in-depth analysis of the failure mechanism of steel-fiber-reinforced concrete.

Yu et al. [25] developed a multiscale modeling approach for concrete structures under dynamic loading. The proposed method combines the macroscale finite element method and the mesoscale discrete element method. The dynamic response of structures was analyzed using the macroscale finite element method; a representative volume element, composed of aggregate, mortar, and interface, was simulated using the mesoscale discrete element method to capture the strain rate effects of concrete. Using the proposed method to predict the cracking of concrete beams, it was found that the crack-closure process is adequately simulated by the proposed method.

Zhou et al. [26] used a discrete element method to establish a three-phase mesoscale model of concrete consisting of mortar, aggregate, and interfacial transition zone. The crushability, real geometry, and random distribution of aggregates were considered by combining 3D scanning techniques and the ball-clump-cluster method. A split-Hopkinson pressure bar device was constructed using the finite difference method, and the interface deformation coordination between the concrete sample and metal rods was implemented via a coupling algorithm. On this basis, the quantitative effects of end-friction on the stress–strain response, dynamic increase factor, damage distribution, and failure mode of concrete were investigated.

Peng et al. [27] proposed a mesoscale model explicitly considering the bond behavior between the randomly distributed steel fibers and matrix of ultra-high performance concrete. The continuous surface cap model was used for mapping the matrix of ultra-high-performance concrete and an algorithm considering the bond-slip effect was employed to model the fiber–matrix interaction. The study was aimed at investigating the dynamic behaviors of concrete members under lateral low-velocity impacts. The model was validated at the material domain level using compression and bending tests and at the member level using drop-hammer tests, giving satisfactory results. 

Zhou and Xu [28] established a 3D mesoscopic model of concrete including mortar, shape-alterable aggregate, and a thickness-controllable interfacial transition zone. Based on the concrete-damaged plasticity method and the cohesive element approach, the analysis was carried out in terms of the macroscopic mechanical response, macroscopic failure morphology, mesoscale fracture propagation, and energy dissipation. Comparing the model with experimental results, it was found to give satisfactory results for both compression tests and failure morphology.

The studies described above show that mesoscale modeling enables the incorporation of internal structural features, which significantly influence the mechanical properties of polymer concrete. Various modeling methods, differing in the method of replicating the individual components of polymer concrete, are used to model its static properties. However, the literature barely mentions the modeling of dynamic properties of epoxy polymer concrete at the mesoscale, especially damping modeling.

In this paper, a novel method of modeling the dynamic properties of polymer concrete is proposed. The method is based on a mesoscale finite element model incorporating aggregates, an interfacial transition zone, and mortar matrix, and the substructural identification method presented in [29]. The dynamic properties of polymer concrete are identified using a model updating method implemented in a substructural identification algorithm. As mesoscale modeling typically provides more complete information about the mechanical behavior of heterogeneous materials compared to purely macroscopic models using homogenized material models, the aim of the study is to develop a mesoscale modeling procedure that will provide a deeper insight into the damping mechanism present in polymer concrete and will provide tools supporting the design of heterogeneous materials, enabling the prediction of their dynamic properties. 

The paper is structured as follows. Section 2 presents the method of mesoscale modeling of polymer concrete dynamic properties along with the substructural identification algorithm. This algorithm, through model updating, makes it possible to determine the parameters of the components of the mesoscale model. In Section 3, the application of the method is presented. It is used to predict the dynamic properties of a polymer concrete beam. This section also includes the validation of the proposed method. This validation is carried out in two stages; the first stage includes an analysis of how the omission of some steps in the proposed methodology affects the accuracy of the results, and in the second stage, the dynamic properties of a polymer concrete beam differing in the proportions of the aggregate fraction are predicted. In Section 4, a discussion of the results obtained is presented. Section 5 contains the final conclusions and summarizes the most important achievements of the study.

## 2. Finite Element Modeling

### 2.1. General Framework

The method starts with a mesoscale finite element model of polymer concrete. This consists of the discretization of the geometric model of heterogenous material reflecting the distribution of individual aggregates in the polymer matrix. Additionally, an interfacial transition zone (*ITZ*) is introduced, which considers the interactions between the aggregates and the matrix. 

After building a mesoscale finite element model, decoupling occurs; as a result, three groups of substructures are formed: (i) polymer matrix, (ii) aggregates, and (iii) *ITZ*. Further, polymer matrix model updating is conducted (local updating stage). The model updating criterion is based on minimizing the differences between numerically and experimentally determined receptances. As a result, the identified parameters of the polymer matrix model are obtained. These parameters do not change in the second stage (i.e., they are treated as known or fixed at a later global updating stage).

Next, the substructures (including updated polymer matrix, aggregates, and *ITZ*) are reassembled into the mesoscale model. Then, global updating takes place, which consists of determining the remaining (not determined in the local updating stage) parameters of the model, i.e., the material parameters of the aggregates and the *ITZ*.

**Figure 1:**
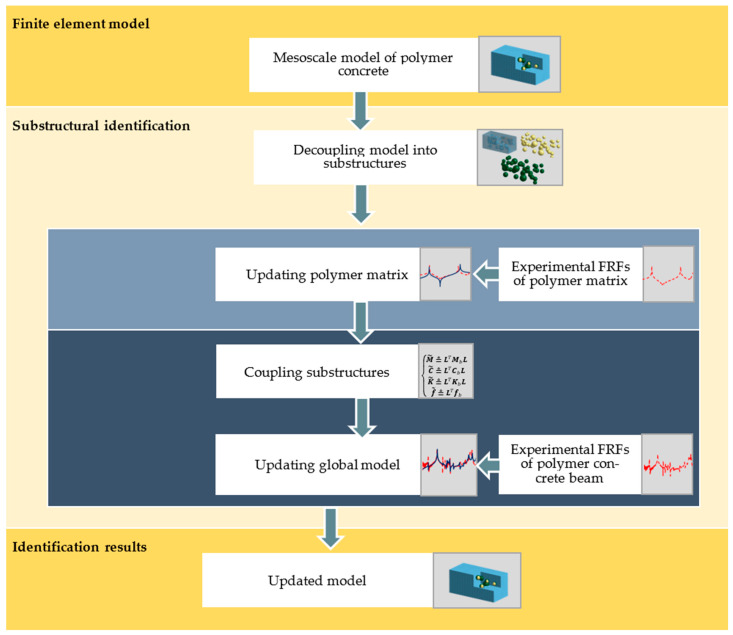
Modeling and model updating workflow.

Summarizing, the use of substructural identification made it possible to determine the parameters of substructures that do not exist in an independent, isolated form like the *ITZ*. Moreover, it allows for determining the amount of damping that *ITZ* brings to the structure. The workflow of the algorithm is depicted in Figure 1.

### 2.2. Finite Element Modeling 

The finite element method is a method of approximating differential equations, which, together with the boundary conditions, constitutes a mathematical model of a certain process or state of a physical system.

In general, the equation of motion for a discrete physical system model takes the following form:

(1) $$M\ddot{u}(t)+C\dot{u}(t)+Ku(t)=f(t)$$

where M, C, K denote the model inertia, damping, and stiffness matrices, respectively; $\ddot{u},\;\dot{u},\;u$ are vectors of generalized accelerations, velocities, and displacements of the model nodes, respectively; f is the vector of forces reduced to model nodes.

**Figure 2:**
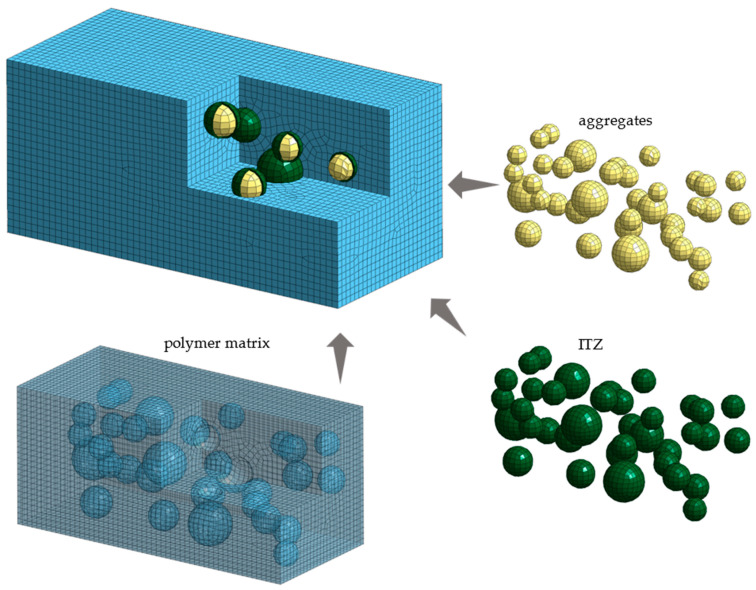
Mesoscale model of polymer concrete.

In the case of mesoscale modeling, the heterogenous material components are divided into three groups: (i) polymer matrix, (ii) aggregates, and (iii) interfacial transition zone. One of the key aspects of mesoscale modeling is the appropriate generation of a geometric model that accurately reflects the proportions of different gradations of aggregates and their mutual positions in the polymer matrix. The pick-and-place method is a widely accepted procedure for generating a geometric model [30]. The exemplary mesoscale model of polymer concrete is depicted in Figure 2. 

### 2.3. Model-Updating Algorithm

According to the presented scheme (Figure 1), in the first step, the finite element model of the analyzed polymer concrete should be decomposed. The decomposition should be carried out in such a way that the individual substructures cover the constituent material domains of the composite structure under analysis, including the interfacial transition zone. This will make it possible to identify the parameters of the constituent substructures and the corresponding components of the polymer concrete. This, in turn, will make it possible to predict the dynamic properties of polymer concretes of similar composition or, alternatively, to extract the properties that aggregates and *ITZ* bring to polymer concrete; that is, elements whose identification as isolated components is practically impossible.

The equation of motion of the substructure finite element model can be written in the form of a system of algebraic equations. This system, in its matrix form, takes the following form:

(2) $$M^{\left(c\right)}{\ddot{u}}^{\left(c\right)}\left(t\right)+C^{\left(c\right)}{\dot{u}}^{\left(c\right)}+K^{\left(c\right)}u^{\left(c\right)}\left(t\right)=f^{\left(c\right)}\left(t\right)$$

where $M^{\left(c\right)}$ is the mass matrix; $C^{\left(c\right)}$ is the damping matrix; $K^{\left(c\right)}$ is the stiffness matrix; ${\ddot{u}}^{\left(c\right)},\;{\dot{u}}^{\left(c\right)},{\;u}^{\left(c\right)}$ are vectors of the degrees of freedom; $f^{\left(c\right)}$ is the external force vector; c is the substructure designation: pm is the polymer matrix, agg denotes the aggregates, and ITZ is the interfacial transition zone.

Next, a frequency response for the substructural model of the polymer matrix is determined. The procedure starts with the general equations of motion and assumes an oscillating load:

(3) $$M^{\left(pm\right)}{\ddot{u}}^{\left(pm\right)}\left(t\right)+C^{\left(pm\right)}{\dot{u}}^{\left(pm\right)}+K^{\left(pm\right)}u^{\left(pm\right)}\left(t\right)=f^{\left(pm\right)}(\omega )e^{i\omega t}$$

where ω is the excitation frequency, $\omega \;\epsilon <\omega_{1},\omega_{p}>$. 

The frequency response in the form of receptance function y(ω) can be found, based on the following dependency:

(4) $$y^{\left(pm\right)}(\omega )=f(\omega ){\left(-\omega^{2}M^{\left(pm\right)}+i\omega C^{\left(pm\right)}+K^{\left(pm\right)}\right)}^{-1},$$

The process of identifying the model parameters (first-stage model updating) is reduced to the task of minimizing the objective function, formulated as follows [31]:

(5) $$Q^{\left(pm\right)}={\left[y^{\left(pm\right),exp}-y^{\left(pm\right),fem}\right]}^{T}\left[y^{\left(pm\right),exp}-y^{\left(pm\right),fem}\right]$$

where $y^{\left(pm\right),exp}$ is the experimentally determined receptance function for the polymer matrix; $y^{\left(pm\right),fem}$ is the receptance for the finite element model of the polymer matrix. The decisive variables in the process of minimization can be Young’s modulus, density, Poisson’s ratio, and a loss factor, which describe the models of the determined substructures.

Later, coupling of the substructure models based on block matrices takes place:

(6) $$M_{b}\ddot{u}(t)+C_{b}\dot{u}(t)+K_{b}u(t)=f_{b}(t)$$

where the individual matrices are defined as follows:

(7) $$M_{b}≜diag\left(M^{\left(pm\right)},M^{\left(agg\right)},M^{\left(ITZ\right)}\right)$$

(8) $$K_{b}≜diag\left(K^{\left(pm\right)},K^{\left(agg\right)},K^{\left(ITZ\right)}\right)$$

(9) $$C_{b}≜diag\left(C^{\left(pm\right)},C^{\left(agg\right)},C^{\left(ITZ\right)}\right)$$

(10) $$f_{b}≜col\left(f^{\left(pm\right)},f^{\left(agg\right)},f^{\left(ITZ\right)}\right)$$

Next, the displacement compliance relationship is defined:

(11) Bu=0

where B is the Boolean matrix, which operates on the interface degrees of freedom and is a signed Boolean matrix if the interface degrees of freedom are matching.

Then, the following dependency is used:

(12) u=Lq

where L is the Boolean matrix localizing the interface degrees of freedom of the substructures in the global dual set of DOF; q is a set of unique interface degrees of freedom. 

The L matrix represents the null space of Boolean matrix B introduced earlier:

(13) L=null(B)

The Boolean localization matrix L transforms the unique set of degrees of freedom to the total set of degrees of freedom u. A detailed description of the formulation of the L and B matrices can be found in [32].

Then, combining Equations (6) and (12), the following equation is obtained:

(14) $$M_{b}L\ddot{q}+C_{b}L\dot{q}+K_{b}Lq=f_{b}$$

Premultiplying Equation (14) by $L^{T}$, the assembled system reduces to:

(15) $$\tilde{M}\ddot{q}+\tilde{C}\dot{q}+\tilde{K}q=\tilde{f}$$

with the primal assembled system matrices defined by:

(16) $$\left\{\begin{array}{c} \tilde{M}≜L^{T}M_{b}L\\ \tilde{C}≜L^{T}C_{b}L\\ \begin{array}{c} \tilde{K}≜L^{T}K_{b}L\\ \tilde{f}≜L^{T}f_{b} \end{array} \end{array}\right.$$

Then, identification of the aggregates and *ITZ* parameters takes place (second-stage model updating). The identified parameters are obtained through minimization of the following function:

(17) $$Q={\left[y^{exp}-y^{fem}\right]}^{T}\left[y^{exp}-y^{fem}\right]$$

where $y^{exp}$ is the experimentally determined receptance function for the polymer concrete beam; $y^{fem}$ is the receptance based on the finite element model of the polymer concrete beam. It should be noted that in the case of the second stage of the model updating, the decision variables should include only the parameters describing the aggregates and interfacial transition zone. Parameters identified for the polymer matrix should be unchanged from those obtained as a result of the first stage of model updating. This will allow determination of the change in the properties introduced into the structure by the aggregates.

## 3. Case Study

### 3.1. Research Object

The case studies concerned a polymer concrete beam, the basic structural component of a machine tool frame that has been presented in [33,34]. The concept of the frame is based on the synergic use of steel (ensuring the assumed stiffness of the structure) and polymer concrete (increasing its ability to dissipate vibration energy). The main challenge is to develop polymer concrete with a proper stiffness and high damping. Therefore, modeling methods are needed to support the material design process.

The selected polymer concrete consisted of an epoxy resin mixed with different grades of mineral filling. Taking the grain size as the dividing criterion, mineral fillings can be categorized as follows: (i) ash, (ii) a fine fraction of grain size 0.1–2 mm (consisting mainly of sand), (iii) a fine fraction of grain size 2–5 mm, (iv) a medium fraction of grain size 5–10 mm, and (v) a coarse fraction of grain size 10–16 mm. The medium and coarse fractions consisted mainly of irregularly shaped gravel. The mass share (percentage) of each fraction in the polymer concrete used in the study is shown in Table 1.

**Figure 3:**
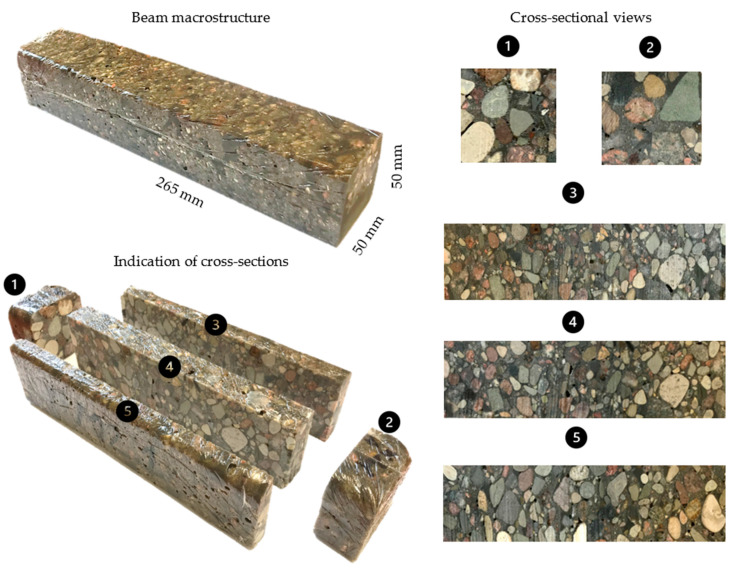
The macrostructure of polymer concrete beams under analysis.

Next, two specimens were made: (i) consisting of polymer concrete and (ii) consisting of polymer matrix material (excluding medium and coarse fractions). The prepared materials were poured into molds with internal dimensions of 50 × 50 × 265 mm and fully vibrated. A sample of the polymer concrete after casting is shown in Figure 3.

The actual beams intended for static and dynamic tests were cut (using water jet cutting) from the inside of the casted beams. The beams to be tested were cut to dimensions of 40 × 40 × 240 mm; before the tests, the beams were cured for 72 h. The choice of such beams was dictated by the desire to obtain objects characterized by low modal density, which allows to avoid the phenomenon of coupling mode shapes. The well-separated resonances allow to precisely identify the material damping, which was one of the goals of the presented study.

### 3.2. Static Tests

**Figure 4:**
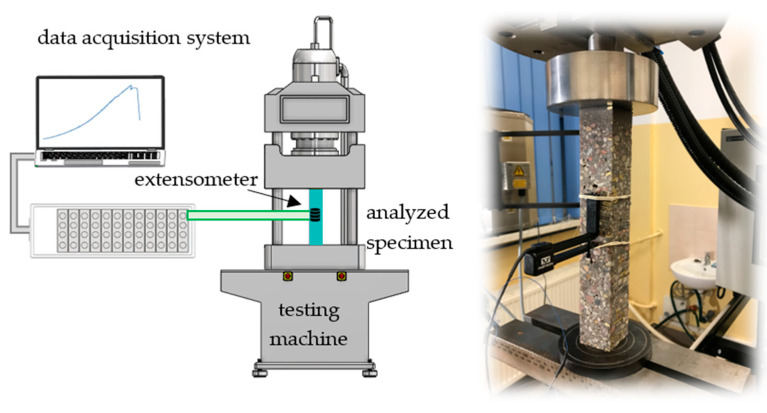
Experimental setup—static tests.

The initial values of the model parameters (moduli of elasticity, Poisson’s ratios, densities) of the polymer concrete matrix were determined based on static tests. Cuboidal specimens of 40 × 40 × 240 mm were subject to compression tests performed on an Instron 8850 (Instron, Norwood, MA, USA) operating in an air-conditioned laboratory at 23 °C and 50% relative humidity. The testing machine was equipped with a class 0.5 measuring head with a range up to 250 kN, and a class 0.2 Instron 2620–601 (Instron, Norwood, MA, USA) tactile extensometer with a 12.5 mm gauge length and a travel of ±5 mm. The test stand is depicted in Figure 4.

The material properties (initial values of the model parameters) with standard uncertainties, supplemented by the value of the loss factor determined on the basis of dynamic tests (using the half-power method and the experimental setup included in Section 3.3), are summarized in Table 2.

### 3.3. Dynamic Tests

To provide the necessary model updating data, an impact test was performed. To minimize the impact of the boundary conditions on the analyzed beam dynamics, the beam was suspended on steel cords (prior testing and analysis were conducted in respect to the influence of different suspension methods’ dynamic properties on the analyzed beam).

**Figure 5:**
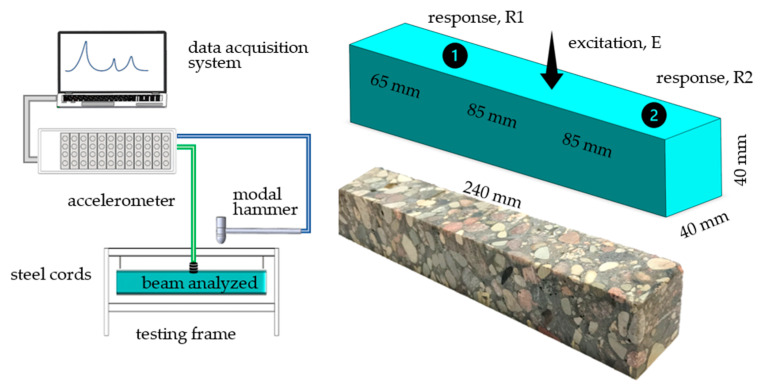
Experimental setup—dynamic tests.

The beam was excited using a PCB 086C01 (PCB Piezotronics, Depew, New York, NY, USA) modal hammer with a steel insert tip and no additional mass. The responses were measured using PCB 356A01 (PCB Piezotronics, Depew, New York, NY, USA) three-axis piezoelectric accelerometers. Data acquisition was performed using Scadas Mobile Vibco and Testlab 2019.1 software (Siemens, Munich, Germany). The test stand is presented in Figure 5 and the signal acquisition parameters are presented in Table 3.

**Figure 6:**
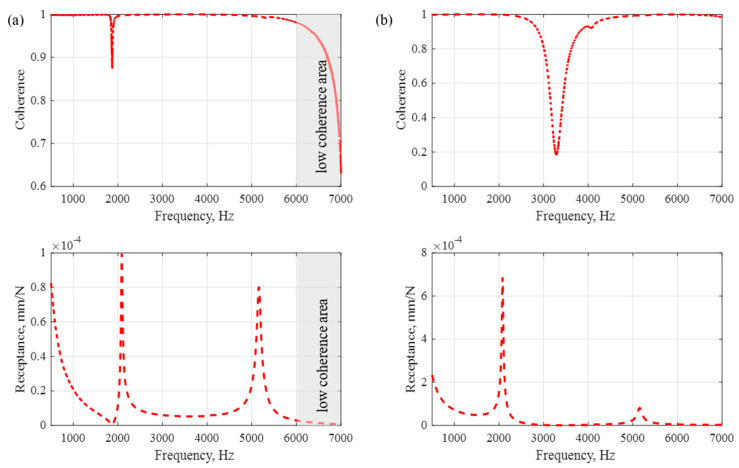
Coherence with experimental frequency response functions for point R1 (**a**) and point R2 (**b**).

The coherence juxtaposed with the frequency responses is shown in Figure 6.

As a result of the analysis of the coherence and frequency response functions, it was decided that the tuning process would be carried out in the range 500–6000 Hz. This was dictated by the low coherence for higher frequencies (area marked in grey), which is clearly visible for point R1 (Figure 6a).

### 3.4. Finite Element Model 

In the first step, a geometric model representing the heterogeneous structure of the polymer concrete was generated. The model was based on spherical models of aggregates and hollow spherical models of the *ITZ* with a wall thickness equal to one-tenth of the diameter of the corresponding aggregates [35]. Aggregates smaller than 5 mm in diameter were incorporated into the polymer matrix [36].

Next, the geometric model was discretized using a Midas NFX 2022 R1 preprocessor (Midas Information Technology Co., Ltd., Seongnam, Republic of Korea) [37]. The calculation area was divided into CHEXA six-sided isoparametric finite elements with eight nodes, and CPENTA five-sided isoparametric elements with six nodes. The applied finite elements were characterized by linear shape functions and three translation degrees of freedom in each node. The process of discretization was supported by the analysis of mesh quality, considering the coefficients of aspect ratio and skewness. The subsequent substructures were modeled as a linear-elastic isotropic material (MAT1). The contact between the substructures was implemented as a coincidence of nodes.

In the next step, a mathematical model of complex stiffness damping was selected to describe the ability of the polymer concrete beam to dissipate the vibration energy. Therefore, the damping matrix C can be expressed as:

(18) C=iηK,

where i is the imaginary unit and η is the loss factor.

**Figure 7:**
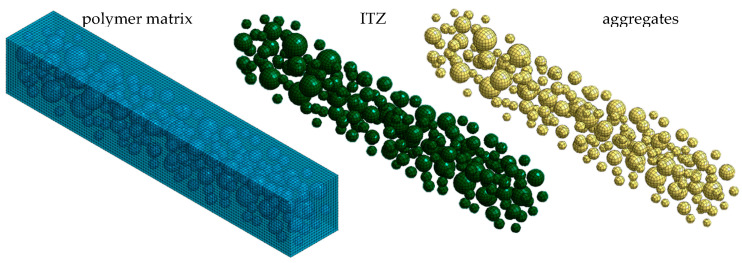
Mesoscale model of the analyzed polymer concrete beam.

In total, the developed model consisted of 161,584 elements and had 246,144 degrees of freedom. The discrete model of a polymer concrete beam is shown in Figure 7.

The models of the polymer matrix, aggregates, and *ITZ* emerged as a result of decoupling. According to the presented algorithm, the first-stage model updating consists of identifying the parameters of a polymer matrix. From a practical point of view, the polymer matrix model updating was conducted on a cuboid specimen with external dimensions corresponding to the beam under consideration. The initial parameters for the model of analyzed polymer concrete matrix were taken from static and dynamic tests, the results of which are shown in Table 2.

The decision variables in the first-stage updating process included only the material properties of the beam. This was dictated by the fact that when building a finite element model, deviations of dimensions and shapes are most often not considered, thus mapping the nominal geometry of the object. To select proper decision variables for the polymer matrix updating, a sensitivity analysis was performed based on the Jacobian introduced in [29]. The analysis included determination of the significance of the influence of individual parameters on the change in the frequency response function. This showed that the parameter with a negligible impact on transverse vibrations is Poisson’s ratio; therefore, it was not included as the decision variable in the updating process. However, considering the fact that this value is pivotal in the accurate representation of torsional mode shapes, Poisson’s ratio was identified in a separate experiment. This consisted of updating the value of the natural frequency corresponding to the torsional mode shape. As a result, the identified Poisson’s ratio was 0.15, and this was set as a constant in the updating of the polymer concrete matrix.

**Figure 8:**
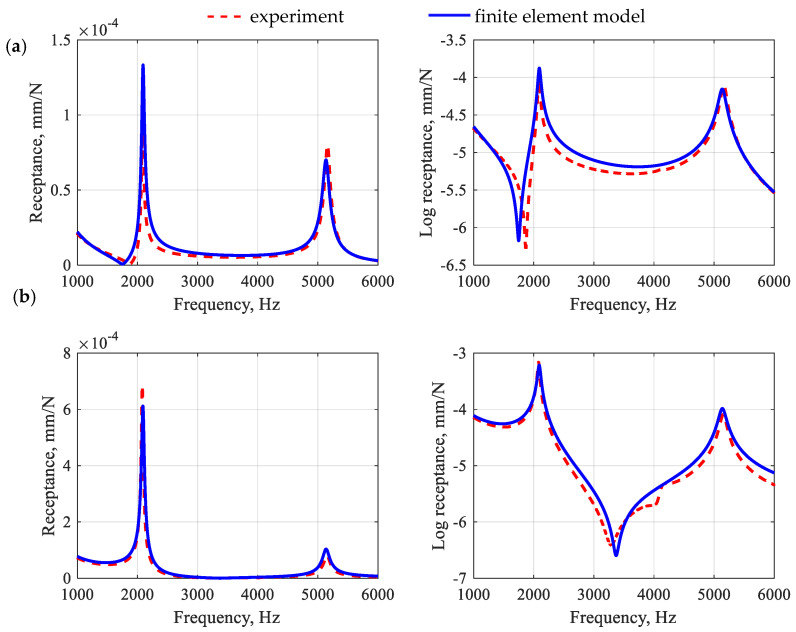
Comparison of frequency response functions for the polymer matrix beam after the first-stage model updating for point R1 (**a**) and point R2 (**b**).

The process of identifying the model parameters was reduced to the task of minimizing the function presented in Equation (5). However, the two frequency response functions were simultaneously incorporated in the updating process: (i) receptance between points E and R1 and (ii) receptance between points E and R2 (Figure 5). To solve the problem, Matlab was implemented and the interior point optimization algorithm was used [38]. Figure 8 shows a comparison of frequency response functions after the first-stage model updating.

When analyzing the accuracy of the mapping of the receptance function for a polymer matrix beam, it can be seen that the model satisfactorily reflects its real counterpart in the qualitative sense. Moreover, the model is characterized by a good ability to reproduce the damping properties of the structure. This is especially evident in the case of the amplitudes of the receptance functions for resonance frequencies. The quantitative comparison (in terms of relative differences between the amplitudes of the receptance function in resonances) shows that for the first two resonances, the relative error values were, respectively, 32.8% and 12.5% for point R1 and 10.8% and 27.5% for point R2, which gives the average error of 20.9%.

**Figure 9:**
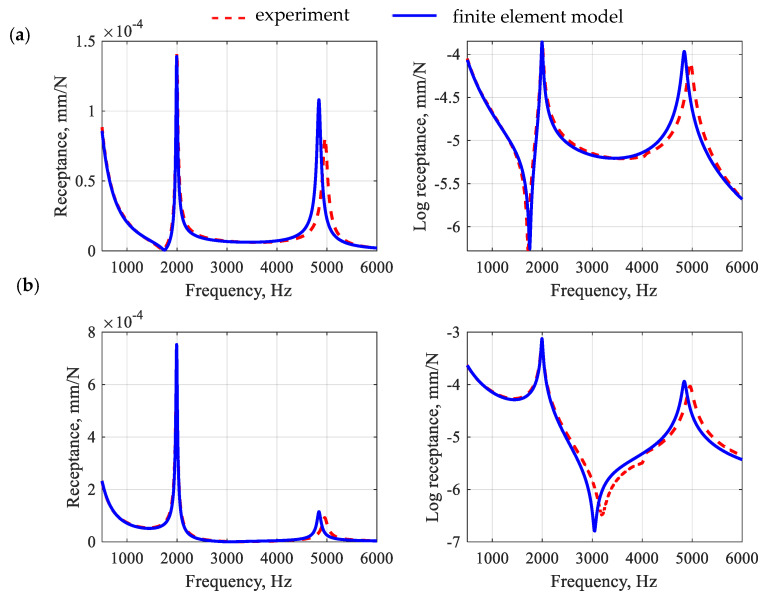
Comparison of frequency response functions for a polymer concrete beam after the second-stage model updating for point R1 (**a**) and point R2 (**b**).

**Figure 10:**
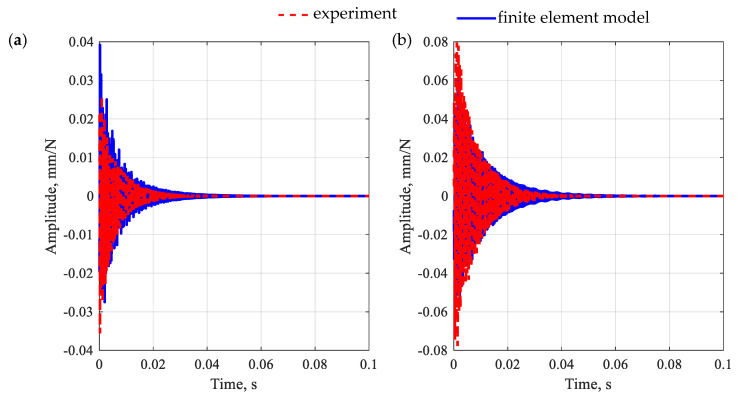
Comparison of impulse response for a polymer concrete beam after the second-stage model updating for point R1 (**a**) and point R2 (**b**).

Next, the second stage of updating took place, which was preceded by a model coupling. In the second stage of updating, the aggregates and *ITZ* parameters were identified (the initial parameters of the aggregates and *ITZ* were taken from [39]). To keep the number of identified parameters low, it was decided to identify single (global) equivalent properties for all aggregates and subsequently all *ITZ*s. Moreover, for the analyzed substructures, the value of Poisson’s ratio was set at 0.15, i.e., corresponding to the identified level for the polymer matrix. Figure 9 shows a comparison of frequency response functions after the second-stage model updating for the polymer concrete beam in question, while Figure 10 shows the impulse response. 

By analyzing the accuracy of the receptance function mapping for a polymer concrete beam, similar conclusions can be drawn as in the case of a polymer matrix beam. The relative differences between the amplitudes of the receptance in the resonances show that for the first two resonances, the relative error values were 2.1% and 31.7% for the point R1 and 7.5% and 24.8% for the point R2, which gives an average error 16.5%.

The observations made for receptances are also reflected in the impulse response. In this case, the model well reproduces both the maximum amplitude of vibrations and their decay time. 

The identified values of the decision variables were obtained and compared with the initial values, as shown in Table 4.

Analyzing the values of the material parameters before and after model updating (provided in Table 4), one can see changes in their values at different levels. The parameter that has changed the most is the loss factor for the *ITZ* $\eta_{ITZ}$ (93.3% change), followed by the loss coefficient for the polymer matrix $\eta_{pm}$ (32.5% change). The accurate identification of those parameters appears to be of key importance when it comes to mapping the damping properties of polymer concrete. Conversely, the smallest change—4.0%—was observed in the case of the aggregate loss factor $\eta_{agg}$. In contrast, accurate identification of Young’s modulus of aggregates $E_{agg}$ appears to have had a significant impact on the mapping of dynamic properties, i.e., a change of 28.6%, translating into a 30 GPa difference in the value that was observed. The smallest changes can be seen for the densities of the individual parameters, so this may indicate a sufficiently accurate determination of the initial values. The relative difference for Poisson’s ratio in all cases is 25%; this is due to the fact that this value was identified in a separate experiment and adopted equally for all model elements.

On the basis of the identified parameters, the natural frequencies and corresponding mode shapes were determined for an unconstrained model of the beam by solving the following eigenproblem:

(19) $$\left(K-\omega_{n}^{2}M\right)\Phi_{n}=0$$

where $\omega_{n}$ is the n-th natural frequency and $\Phi_{n}$ is the n-th mode shape vector. Calculations were performed using Nastran Solver (SOL103).

Next, the experimental modal analysis was conducted using the test stand depicted in Figure 5. The beam was excited in three perpendicular directions and the responses were measured at 56 points. As a result of the impulse test conducted, 168 frequency response functions were determined. 

Further, using the Polymax algorithm [40,41], the parameters of the modal models of the beams analyzed were estimated. The obtained modal models were validated using the MAC criterion, eliminating interdependent vectors in the mode shape (the limit value of 10% was assumed) [42]. The experimental verification of natural frequencies for the initial and updated parameters is shown in Table 5. The value of the relative error was used as the measure of the agreement of the natural frequencies of the finite element model with the experimentally determined natural frequencies. 

Analyzing the data presented in Table 5, it can be seen that in the case of the polymer matrix beam, compliance of the first five modes was obtained. Relative differences for natural frequencies do not exceed 0.3% and are 0.2% on average. This is a significantly better result than in the case of a beam model built based on the values of material properties determined in static tests (maximum error 1.4%, on average 1.2%).

A similar situation can be observed for a polymer concrete beam; the maximum error does not exceed 2.3% and is 1.2% on average. Relative error values before updating reach a maximum of 7.1% and an average of 5.9%. The level of accuracy of the presented results is at a similar level as in [43,44,45], which presented macroscale models.

### 3.5. Finite Element Model Validation 

The validation of the developed finite element model, and, thus, the proposed modeling method, was carried out in two steps. The first step was to investigate how deviations from the proposed procedure affect the prediction of the dynamic properties of the analyzed beam. The second step was to predict the dynamic properties of another beam that differs in aggregate fraction proportions.

**Figure 11:**
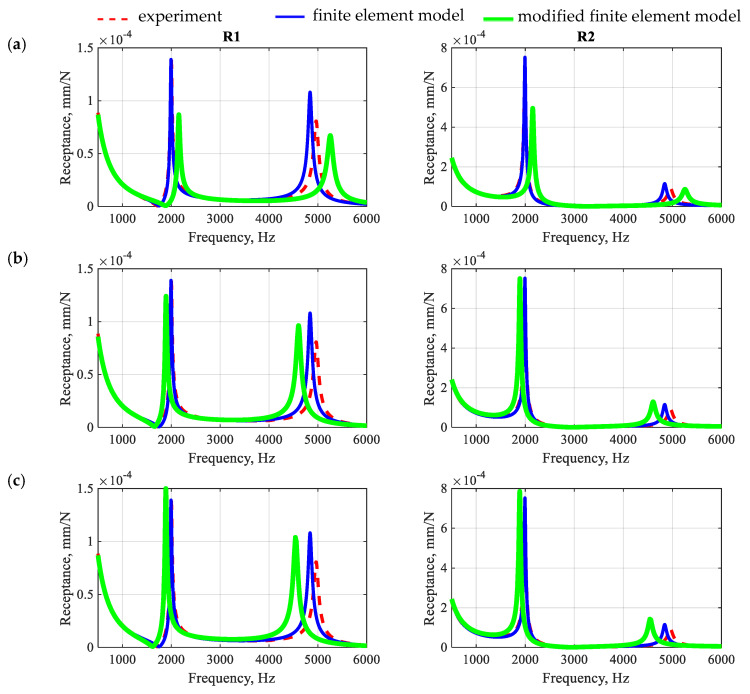
Comparison of the numerically and experimentally determined receptance functions determined between point E and points R1 and R2 for model 1 (**a**), model 2 (**b**), and model 3 (**c**).

As stated above, the first step was to investigate how derogations from the proposed procedure influence the accuracy of prediction of the dynamic properties of the analyzed beam. Therefore, two separate analyses were carried out: (i) the beam was modeled with incorrect mapping of the filling fraction proportions, and aggregates smaller than 8 mm in diameter were incorporated into the polymer matrix (model 1), and (ii) the beam was modeled excluding the model updating stages (model 2—no polymer matrix properties considered in updating process, and model 3—no aggregates and *ITZ* updating). The results are depicted in Figure 11.

The obtained receptances show that deviations from the proposed modeling methodology cause deterioration of the accuracy of the obtained results. It seems that the wrong mapping of the proportions of the filling fractions most significantly affects the accuracy of the results, both in terms of mapping the resonance frequencies and the amplitudes of the receptance function.

Omission of the identification of the polymer matrix has a smaller impact on the accuracy of mapping the properties. However, such an approach seems to distort the structure of the model, which can be seen by changing the ratios of the individual amplitudes of the receptance function.

The smallest differences result from omitting the identification of aggregates and the *ITZ*. This, however, will largely depend on the assumed initial values for these substructures. In summary, both accurate modeling of the geometry and accurate identification of certain substructures have an impact on the modeling accuracy. High accuracy and unambiguity of the results is particularly important in the case of research aimed at explaining the dynamic phenomena (including the damping mechanism) occurring in polymer concrete.

**Figure 12:**
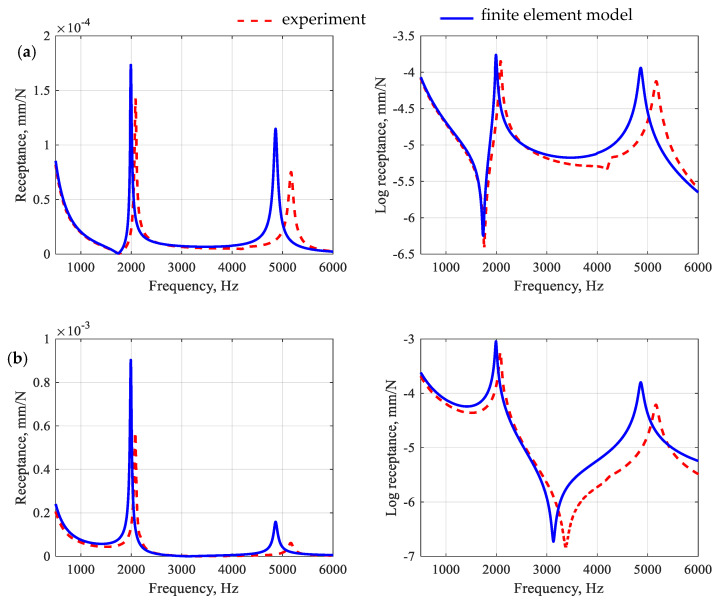
Comparison of frequency response functions for a modified polymer concrete beam for point R1 (**a**) and point R2 (**b**).

**Figure 13:**
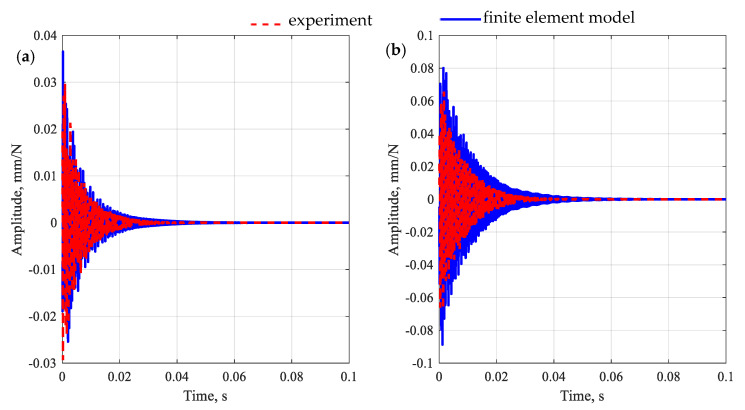
Comparison of impulse response functions for a modified polymer concrete beam for point R1 (**a**) and point R2 (**b**).

In the second stage of validation, the beam was made from a polymer concrete differing in aggregate fraction proportions (medium fraction 20% and coarse fraction 33%). For the developed model, the frequency response functions, impulse responses, and modal models were determined numerically and compared with experimental results. The comparison of the selected receptance functions is depicted in Figure 12 and the impulse response is shown in Figure 13. The modal models are compared in Table 6.

By analyzing the accuracy of the receptance function mapping for a modified polymer concrete beam, the following relative differences between the amplitudes of the receptances were observed: 18.3% and 55.8% for the point R1 and 58.2% and 154.3% for the point R2, which gives an average error 71.7%. The observations made for the receptors are also reflected in the impulse response. In this case, the model satisfactorily reproduces both the maximum vibration amplitude and its decay time.

The validation of the model showed that the identified parameters describing the polymer matrix, aggregates, and *ITZ* satisfactorily allow predicting the dynamic properties of a beam made of polymer concrete with a modified composition. In the analyzed frequency range, full compliance of the mode shapes was achieved, with the maximum relative error for natural frequencies at the level of 4.7% (for the torsional mode), and an average of 3.8%. In addition, a fair representation of the frequency response functions and the impulse response was obtained.

## 4. Discussion 

The proposed modeling method allowed for accurate mapping of the dynamic properties of the polymer concrete beam. The obtained results show that all five modes in the analyzed frequency range were successfully reproduced by the model. The average relative error obtained for the natural frequencies was 1.2% and the maximum error did not exceed 2.3%. In addition, high-accuracy frequency response functions were obtained, as well as impulse response functions.

The applied substructural identification method allowed the maximum relative error to decrease (with respect to the initial parameters model) for natural frequencies from 7.1% to 2.3%, and from 5.9% to 1.2% on average. The validity of the applied updating is also manifested in the case of receptance functions.

The analysis of the model-updating results provides some guidance on the identification of model parameters for each material group. More precisely, the analysis shows that the largest changes were in the loss factor values for the polymer matrix and *ITZ*, i.e., relative differences of 32.5% and 93.3, respectively. These results can be interpreted in two ways: (i) the identification of initial values based on representative material samples may not be sufficient to accurately represent the dynamic properties of the composite material, and (ii) a far-reaching conclusion, or more of a hypothesis, suggests that these parameters may have a key influence on the damping of polymer concrete. However, this aspect requires further research. A similar observation, albeit characterized by a smaller change (28.6% relative difference) was made for the Young’s modulus values of the aggregates.

The proposed method was validated using the identified parameters of the polymer matrix, aggregates, and *ITZ* to anticipate the dynamic properties of a beam made of polymer concrete differing in aggregate fraction proportions. The obtained results were satisfactory, i.e., the maximum relative error obtained for natural frequencies was 4.7% (for the torsional mode), with an average of 3.8%. Additionally, fair mapping of the receptance function and the impulse response was achieved. 

The equivalent loss factor, determined globally for a polymer concrete beam, gives a result very similar to that obtained in [29]. Herein, the value was 0.0145, compared to 0.0152 obtained in [24], which gives a relative difference of 4.6%.

A potential limitation of the presented algorithm may be the structural inconsistency of the model (expressed in the incompatibility of torsional mode shapes). Such a situation would require deeper consideration of the model and its possible modification. One of the possible directions for improving the results is the development of a mesoscopic model based on realistic aggregate models for which it is possible to define their specific orientation [46]. 

Another possible limitation may also apply to the modeling of materials with micro-reinforcement, e.g., in the form of steel fibers; here, it would also be necessary to modify the geometry generator. Furthermore, it would probably be necessary to introduce a separate *ITZ* for micro-reinforcement, although this requires further research.

## 5. Conclusions

This study addressed the problem of modeling the dynamic properties of highly heterogeneous materials, such as the considered polymer concrete. Mesoscale finite element modeling was used with substructural identification to predict the dynamics of polymer concrete beams. The proposed modeling methodology enables fair mapping, as well as the prediction of modal models, frequency response functions, and impulse responses. Moreover, it can be used to estimate the dynamic properties of materials of similar composition. Thus, to a limited extent, it can serve as a tool supporting the design of materials.

Although the work does not explain the nature of damping occurring in polymer concrete, it does provide tools for modeling dynamic properties in a vast frequency range and extracting certain components’ parameters. It also provides important insights into modeling aspects and the rationale for future work. These should include the development of an advanced geometry generator, the work for which will be supported by the tomography of specimens in order to accurately map the distribution of aggregates in the matrix. 

The substructural identification method used gives some indication of the accuracy of determining the initial parameters of certain material components. According to the results obtained, the largest changes after updating were observed for the parameters describing the damping of the polymer matrix and *ITZ*, which sets the direction for further research to explore these aspects.

One of the next steps may be to carry out the indentation of a single aggregate into a polymeric matrix, which should give a better insight into the damping mechanism present at the interfacial transition zone. Such studies should enrich the current state-of-the-art, constituting another step towards explaining the nature of the damping of heterogeneous materials such as the analyzed polymer concrete.

## Figures and Tables

**Table 1:** Composition of the applied polymer concrete.

	Epoxy Resin	Ash	Sand (0.1–2 mm)	Fine Fraction (2–5 mm)	Medium Fraction (5–10 mm)	Coarse Fraction (10–16 mm)
Polymer concrete	15%	1%	16%	15%	35%	18%

**Table 2:** Material properties of steel and polymer matrix determined based on the experimental study.

Property	Polymer Concrete Matrix
Modulus of elasticity, $E_{pm}$	20.0 ± 0.5 GPa
Poisson’s ratio, $\upsilon_{pm}$	0.20 ± 0.05
Density, $\rho_{pm}$	2050 ± 6 kg/m³
Loss factor, $\eta_{pm}$	0.0166

**Table 3:** Parameters of signal acquisition.

Parameter	Value
Bandwidth	16,384 Hz
Frequency resolution	0.5 Hz
Signal acquisition time	2 s
Frequency response function estimator	H_1_
Number of averages	10
Scaling of the frequency response function	global

**Table 4:** Comparison of the decision variable values before and after the model updating.

Property	Initial Value	Identified Value	Relative Difference
Polymer matrix			
Modulus of elasticity, $E_{pm}$	20.0 GPa	19.2 GPa	8.6%
Poisson’s ratio, $\upsilon_{pm}$	0.20	0.15	25.0%
Mass density, $\rho_{pm}$	2050 kg/m³	1922 kg/m³	6.2%
Loss factor, $\eta_{pm}$	0.01660	0.02200	32.5%
Aggregates			
Modulus of elasticity, $E_{agg}$	70.0 GPa	90.0 GPa	28.6%
Poisson’s ratio, $\upsilon_{agg}$	0.20	0.15	25.0%
Mass density, $\rho_{agg}$	2500 kg/m³	2430 kg/m³	2.8%
Loss factor, $\eta_{agg}$	0.00150	0.00156	4.0%
*ITZ*			
Modulus of elasticity, $E_{ITZ}$	70.0 GPa	60.0 GPa	14.3%
Poisson’s ratio, $\upsilon_{ITZ}$	0.20	0.15	25.0%
Mass density, $\rho_{ITZ}$	2500 kg/m³	2330 kg/m³	6.8%
Loss factor, $\eta_{ITZ}$	0.00150	0.00010	93.3%

**Table 5:** The results of experimental verification of the natural frequencies of the model.

Mode Type	Experimental Study	FEM Model (Initial Parameters)	Relative Error δ	FEM Model (Updated Parameters)	Relative Error δ
Polymer matrix beam					
1st bending	2090 Hz	2120 Hz	1.4%	2097 Hz	0.3%
1st bending	2093 Hz	2120 Hz	1.3%	2097 Hz	0.2%
1st torsional	4108 Hz	4072 Hz	0.9%	4110 Hz	0.1%
2nd bending	5162 Hz	5223 Hz	1.2%	5176 Hz	0.3%
2nd bending	5165 Hz	5223 Hz	1.1%	5176 Hz	0.2%
		**On average:**	1.2%	**On average:**	0.2%
Polymer concrete beam					
1st bending	1998 Hz	1889 Hz	5.4%	1994 Hz	0.2%
1st bending	2005 Hz	1901 Hz	5.2%	2001 Hz	0.2%
1st torsional	4016 Hz	3801 Hz	5.4%	3921 Hz	2.3%
2nd bending	4962 Hz	4607 Hz	7.1%	4861 Hz	2.0%
2nd bending	4965 Hz	4630 Hz	6.7%	4899 Hz	1.3%
		**On average:**	5.9%	**On average:**	1.2%

**Table 6:** The results of experimental validation of the natural frequencies of the model.

Mode Type	Experimental Study	FEM Model	Relative Error δ
1st bending	2074 Hz	1991 Hz	4.0%
1st bending	2076 Hz	1998 Hz	3.8%
1st torsional	4192 Hz	3992 Hz	4.7%
2nd bending	4812 Hz	4863 Hz	1.1%
2nd bending	5171 Hz	4902 Hz	3.8%
		**On average:**	3.8%

## Data Availability

The author confirm that the data supporting the findings of this study are available within the article.
